# Public health implications of complex emergencies and natural disasters

**DOI:** 10.1186/s13031-017-0135-8

**Published:** 2017-11-29

**Authors:** Amanda Culver, Roger Rochat, Susan T. Cookson

**Affiliations:** 1 Rollins School of Public Health, Emory University, Atlanta, GA USA; 2 Center for Global Health, Centers for Disease Control and Prevention, 1600 Clifton Rd, NE, Mailstop E-22, Atlanta, GA 30329 USA

**Keywords:** Complex emergencies, Natural disasters, Outbreaks, Vaccine-preventable diseases

## Abstract

**Background:**

During the last decade, conflict or natural disasters have displaced unprecedented numbers of persons. This leads to conditions prone to outbreaks that imperil the health of displaced persons and threaten global health security. Past literature has minimally examined the association of communicable disease outbreaks with complex emergencies (CEs) and natural disasters (NDs).

**Methods:**

To examine this association, we identified CEs and NDs using publicly available datasets from the Center for Research on the Epidemiology of Disasters and United Nations Flash and Consolidated Appeals archive for 2005–2014. We identified outbreaks from World Health Organization archives. We compared findings to identify overlap of outbreaks, including their types (whether or not of a vaccine-preventable disease), and emergency event types (CE, ND, or Both) by country and year using descriptive statistics and measure of association.

**Results:**

There were 167 CEs, 912 NDs, 118 events linked to ‘Both’ types of emergencies, and 384 outbreaks. Of CEs, 43% were associated with an outbreak; 24% NDs were associated with an outbreak; and 36% of ‘Both’ types of emergencies were associated with an outbreak. Africa was disproportionately affected, where 67% of total CEs, 67% of ‘Both’ events (CE and ND), and 46% of all outbreaks occurred for the study period. The odds ratio of a vaccine-preventable outbreak occurring in a CE versus an ND was 4.14 (95% confidence limits 1.9, 9.4).

**Conclusions:**

CEs had greater odds of being associated with outbreaks compared with NDs. Moreover, CEs had high odds of a vaccine-preventable disease causing that outbreak. Focusing on better vaccine coverage could reduce CE-associated morbidity and mortality by preventing outbreaks from spreading.

## Background

The last decade has seen a record-high number of displaced persons globally [1]. For 2016, the United Nations High Commissioner for Refugees (UNHCR) estimates that 65.6 million fled their homes because of conflict, violence, persecution, or human rights violations [1]. Persons fleeing because of natural disasters (NDs) has further increased the numbers. The Internal Displacement Monitoring Center estimates that NDs displaced 24.2 million persons in 2016 [2].

A Complex Emergency (CE) is “a humanitarian crisis which occurs in a country, region, or society where there is a total or considerable breakdown of authority resulting from civil conflict and/or foreign aggression” [3]. This crisis can result in high morbidity and mortality among the affected persons. Infectious diseases play a significant role in the CE-associated morbidity and mortality; in one case, up to 95% of recorded deaths [4]. Reasons that CEs put populations at risk are because of large-scale displacement and crowding, lack of clean water, poor sanitation, malnutrition, and low health care and vaccine coverage [4, 5]. Additionally, they may last for years leading to long-term deprivation of basic and health services (e.g., Sudan’s conflict since the 1980’s [6]). Finally, large-scale displacement may expose populations to diseases to which they have no immunity or conversely, they may introduce diseases, like malaria, into previously non-endemic areas [7].

A ND is a phenomenon caused by rapid or slow onset of geophysical, hydrological, climatological, or meteorological events [8]. People have long associated NDs with a high risk for outbreaks. While this belief persists, evidence is lacking, except for specific cases such as Ebola or when NDs lead to similar circumstances as CEs [9, 10].

CEs and NDs may not always occur independently. One occurrence can precede another or they may occur concurrently (e.g., Somalia’s conflict was exacerbated by drought). These emergencies can occur with outbreaks, or outbreaks may occur with no emergency. A study from 1995 to 2004, found displaced persons, especially those involved in CEs, were at increased risk of infectious disease outbreaks [5]; such outbreaks can spread and can threaten global health security.

This research has two aims: 1) for 2005–2014, analyze the number and overlap of CEs, NDs, and outbreaks and 2) by determining the types of outbreaks, whether vaccine-preventable or not, identify the public health implications of these data and their potential to impact planning, response, and interventions.

## Methods

We reviewed all data sources for the study period 2005–2014 and a single reviewer examined each individually to ascertain the nature of the emergency or outbreak and the number affected.

The CEs were identified using two primary sources: Center for Research on the Epidemiology of Disasters (CRED) Emergency Event Database (CE-DAT) and United Nations Office for the Coordination of Humanitarian Affairs (OCHA) Consolidated and Flash Appeal archives [11, 12]. We used the third source, UNHCR archives and records, for verification. Inclusion criteria for CE-DAT surveys were meeting all three of the following: 1) CE threshold crude mortality rates (>1/10,000/day), 2) country-specific (as opposed to regional) appeals, and 3) ≥10,000 affected persons.

We queried the CE-DAT database, which stores nutrition and mortality survey data of CE-affected countries, for 2005–2014 using the *Tables* function on the website. Each country in the database was queried independently using search criteria of 1) country name, all available *Admin Div. 1* (referring to all available provinces or districts), and 2) crude mortality rate (CMR). All returned entries were exported and organized in Microsoft Excel (Microsoft©, Redmond, WA). CRED acknowledges that this database has inherent limitations given surveys are submitted voluntarily, so gaps in reporting may exist and their validity is a concern, as the submitted surveys are not compared with raw data [13]. For these reasons, we used the secondary source of CEs.

OCHA maintains a Central Emergency Response Fund (CERF) to which countries can appeal for rapid humanitarian response and funding to support ND- or armed conflict-affected populations [14]. In order to receive assistance, countries must file with OCHA a request called a Flash or Consolidated Appeal. We searched, collected and organized appeals in Microsoft Excel by country and year, 2005–2014. We examined the attached PDF copies of the original appeal archive filed documents for the number affected and categorized as armed conflict, natural disaster, or other.

We cross-referenced CE-DAT surveys and OCHA appeals by year and country to identify dual reporting of the same emergency. If observations appeared the same, we recorded them as one event. If observations were clearly different emergencies, we recorded them as separate events and verified them using the tertiary source, the UNHCR archives and records, using yearly country reports to confirm emergency and number affected. Additionally, we used UNHCR country reports to verify number affected in all CE-DAT surveys.

The NDs were identified using the CRED International Disaster Database (EM-DAT) and again the OCHA Flash and Consolidated Appeal archives [8, 12]. Our inclusion criteria for NDs were 1) country-specific appeals and 2) affecting ≥10,000 persons. We used the *Database, Advanced Search* function on the website to query an EM-DAT database for 1) *Period*: 2005–2014, 2) *Country*: all included, 3) *Disaster Classification*: Natural. We exported and organized all entries in Microsoft Excel. We used the EM-DAT as it is widely cited in policy documents and research analyses by organizations, such as International Monetary Fund and Oxfam, as a source of NDs [15–17]. We collected ND Flash and Consolidated appeals in the same way as for CE flash and consolidated appeals and identified countries with ongoing disasters in the year(s) following the original event. We categorized NDs into six categories: drought, earthquake, extreme temperature, flood, storm, and other. ‘Other’ included landslides, wildfires, and volcanic activity but grouped together because of their infrequent occurrences.

For CEs and NDs spanning multiple years, we counted each year as one occurrence to ensure that no type of emergency would have a greater chance of being associated with an outbreak. We did this because especially for CEs, which tend to last a longer time than NDs, there would be no lesser or greater chance of association with an outbreak. Additionally, we counted only once when multiple reports for the same country starting in the same year involved the same type of emergency. For example, the 2010 Haiti earthquake appeals occurred in 2010 and the three subsequent years but because of extensive earthquake-related infrastructure damage and ongoing displacement crisis, we counted the flash appeals and ND as separate annual occurrences.

We collected infectious disease outbreaks from the WHO disease outbreak archive and categorized them by country, numbers affected, and disease [18]. This archive contains all outbreaks reported to the WHO from national International Health Regulation (IHR) officers, usually within the Ministry of Health. For outbreaks spanning multiple years, we counted each year as a separate occurrence. Because the 2009 H1N1 pandemic caused many outbreaks and affected many people, we also analyzed data excluding H1N1. We calculated Case Fatality Rates (CFRs) and outbreaks with CFR >70% were classified into total outbreaks, outbreaks excluding H1N1, and outbreaks affecting ≥100 persons, to control for diseases with very low infection numbers but high mortality rates (i.e., avian influenza and Ebola). Because vaccine-preventable diseases (VPDs) have the clearest implications for public health interventions, we analyzed these outbreaks individually.

We cross-referenced CEs and NDs and, if both occurred in the same year, we categorized them as ‘Both’ and removed them from the individual categories. We then cross-referenced these three separate categories (CE, ND, Both) with the WHO outbreak archive and linked emergencies that occurred in the same year as an outbreak.

Once outbreaks were linked to emergencies (CEs, NDs, or Both) or not (associated with no emergency), basic descriptive statistics for emergency type and geographic region were performed on numbers and proportion of outbreaks. We calculated bivariate odds ratios (OR) and 95% confidence limits (CL) using chi-square test in OpenEpi (AG Dean, KM Sullivan, MM Soe, Atlanta, GA) software comparing outbreaks (total, or vaccine-preventable or not), with and without H1N1 outbreaks, with each type of emergency and the region of the emergency. A *P* value of <0.05 was considered significant.

## Results

### Complex humanitarian emergencies

CE-DAT database query yielded 1554 surveys; 1008 were excluded because CMR was ≤1 death/10,000/day and 458 were duplicates, yielding 88 CEs. The Flash and Consolidated Appeals archive yielded 249 documents with 72 categorized as NDs, 23 excluded for falling into category ‘other’ (poverty and food insecurity), 4 duplicates, 12 documents removed for being regional reports only, and 7 could not be verified in the UNHCR database, yielding 131 CEs Flash appeals. We examined CE-DAT and flash appeals data for overlap. The combined CE-DAT and appeals data without duplicates yielded 167 total CEs between 2005 and 2014 that were verified using UNHCR country report documents.

The 167 CEs occurred in 42 countries, with most in Africa (*n* = 112, 67%), followed by the Middle East (*n* = 36, 22%) (Table 1). While Africa suffered the largest number of CEs, the CEs with the largest number of affected population were in the Middle East, which had eight of the 10 largest events by number of affected people. Overall, the mean number of persons affected per individual CE was 1,102,780.

**Table 1:** Populations affected by Complex Emergencies (CEs) and Natural Disasters (NDs), 2005–2014

Region	Number of CEs (% of total)	Population affected by CEs	Number of NDs (% of total)	Population affected by NDs
Africa	112 (67%)	78.9 million	267 (29%)	495 million
Asia	18 (11%)	12.8 million	294 (32%)	558 million
Middle East	36 (22%)	94.2 million	46 (5%)	135 million
East. Europe	1 (1%)	400,000	30 (3%)	137 million
Americas and Caribbean	0	0	234 (26%)	332 million
West Europe and Oceania	0	0	41 (4%)	72 million
Globally	167		912	

### Natural disasters

The EM-DAT database query yielded 1998 events between 2005 and 2014 with 1103 excluded because they affected fewer than 10,000 persons, resulting in 895 observations. When we compared these with the 72 flash appeals for NDs, we found no new events. We included seventeen additional events representing ongoing years of appeal related to prior events, resulting in 912 NDs between 2005 and 2014.

NDs occurred in 139 different countries. Regionally, Asia (32%) and Africa (29%) had the most NDs in the study period (Table 1). Nine of the 10 largest NDs occurred in China. Overall, the most commonly occurring events were floods (53%), storms (17%), and droughts (14%) (Table 2). The mean number of persons affected by all NDs was 1,935,519.

**Table 2:** Natural Disasters by type and year, 2004–2015

Year	Extreme Temperature	Other	Earthquake	Drought	Storm	Flood
2005	0	3	6	15	17	35
2006	1	10	7	6	10	43
2007	2	5	4	8	19	59
2008	3	2	5	16	18	53
2009	4	4	7	13	14	48
2010	3	9	7	10	20	55
2011	7	1	10	13	11	44
2012	7	1	9	18	20	51
2013	5	3	7	8	16	54
2014	5	4	9	16	12	40
Total	37	42	71	123	157	482
Median	4	4	7	13	17	50
Percent of Total	4.1%	4.6%	7.8%	13.5%	17.2%	52.9%

### Both complex humanitarian emergencies and natural disasters

When we compared CE and ND databases, 118 observations fell into the ‘Both’ category, meaning an ND and CE occurred in the same country in the same year. Thus, we classified 84 events as “CEs only” and 795 as “NDs only.”

The 118 ‘Both’ events occurred in 29 countries: Africa (67%), Asia (20%), and the Middle East (14%). Seven of the 10 largest events by population affected in the ‘Both’ category were African droughts. The mean number of persons affected was 1,994,785.

### Infectious disease outbreaks

The WHO outbreak archive yielded 963 reports; of those, 355 were new outbreak reports; the remaining were updates. After separating ongoing occurrences by year, we identified 29 additional outbreaks resulting in 384 outbreaks during 2005–2014.

The average annual number of outbreaks was 38 (median 29, range 24–124). The 2009 H1N1 pandemic resulted in largest number of annual outbreaks, accounting for 104. Excluding the 2009 H1N1 outbreaks, the annual average was 28 (median 28, range 20–37). The mean number of persons affected was 5867; excluding the 2009 H1N1 epidemic, it was 7758.

Examining the outbreak distribution by region, 46% occurred in Africa and 17% in Asia, with all other regions accounting for <10% of outbreaks each. The 10 outbreaks with the highest number infected occurred in Asia, Africa, South America, and North America (Table 3), the latter region because of H1N1. Of these 10 outbreaks, VPDs caused seven. Besides avian influenza outbreaks of 1–8 cases with 100% CFR (*n* = 7), other diseases with the highest CFR regardless of case counts among these 20 top outbreaks, occurred in Europe, Africa, Asia, and the Middle East and included viral hemorrhagic fevers (*n* = 4), Middle Eastern Respiratory Syndrome (MERS-CoV, *n* = 3), yellow fever (*n* = 5) and poliomyelitis (*n* = 1). Of the 10 outbreaks with highest CFRs affecting ≥100 persons, eight occurred in Africa and included Ebola, poliomyelitis, and Rift Valley fever (Table 4).

**Table 3:** Ten outbreaks with the highest number affected per outbreak by country and disease, 2005–2014

Date	Country	Region	Disease	Total infected	Total mortality	Case fatality rate
2006	India	Asia	Chikungunya	1,250,000	Unknown	Unknown
2005	Guinea Bissau	Africa	Cholera	143,030	252	0.18%
2008	Brazil	South America	Dengue	120,570	48	0.04%
2011	Democratic Republic of Congo	Africa	Measles	103,000	1100	1.07%
2009	Zimbabwe	Africa	Cholera	71,927	2758	3.83%
2010	Haiti	Caribbean	Cholera	60,240	1415	2.35%
2006	Angola	Africa	Cholera	43,076	1642	3.81%
2007	Iraq	Middle East	Cholera	30,000	14	0.05%
2010	Nigeria	Africa	Cholera	29,115	1191	4.09%
2009	United States	North America	H1N1	27,717	127	0.46%

**Table 4:** Ten outbreaks having 100 or more infected with highest case fatality rates by country and disease, 2005–2014

Date	Country	Disease	Total Infected	Total Mortality	Case Fatality Rate
2014	Guinea	Ebola	607	406	67%
2014	Liberia	Ebola	1082	624	58%
2007	Viet Nam	Avian Influenza	101	47	47%
2010	Democratic Republic of Congo	Poliomyelitis	184	85	46%
2006	Somalia	Rift Valley Fever	114	51	45%
2007	Democratic Republic of Congo	Ebola	372	166	45%
2013	Saudi Arabia	MERS-CoV^a^	160	69	43%
2014	Sierra Leone	Ebola	910	392	43%
2007	Tanzania	Rift Valley Fever	264	109	41%
2007	Sudan	Rift Valley Fever	601	211	35%

^a^ *MERS-CoV* Middle Eastern Respiratory Syndrome - Coronavirus

In terms of type of outbreak, 42% were acute respiratory illnesses (influenza, MERS-CoV) followed by the VPDs (39%): yellow fever (10.0%), poliomyelitis (9.1%), meningococcal disease (8.7%), and cholera (7.8%). This proportion was similar for all regions, except Africa where 70% of the outbreaks were vaccine preventable.

Of the 84 ‘CEs only’ events, 36 (43%) were linked with outbreaks. Of the 795 ‘ND only’ events, 191 (24%) were linked with outbreaks. Of the 118 ‘Both’ events, 42 (36%) were linked with outbreaks. In bivariate analysis, the odds ratio (OR) of any outbreak occurring in a CE only versus an ND only was 2.42 (95% CL 1.51, 3.84). The OR of any outbreak occurring in a ‘Both’ emergency setting versus a CE only setting was 0.72 (95% CL 0.41, 1.29), and versus an ND only setting was 1.74 (95% CL 1.15, 2.62) (Table 5). Excluding H1N1 outbreaks, the OR of an outbreak occurring in a CE only versus an ND only was 2.27 (95% CL 1.57, 3.25) (Table 5). Stratifying regionally revealed no significant relationships, except Africa, where the OR of an outbreak occurring in a CE only versus an ND only were 3.50 (95% CL 2.12, 5.84).

**Table 5:** Bivariate analysis of association of outbreaks overall or whether of vaccine-preventable disease (VPD) with type of emergency, complex emergency (CE) or natural disaster (ND), 2005–2014

Event	Odds Ratio	95% confidence limits	Chi squared	*P* value
Including 2009 H1N1 outbreaks				
Outbreak occurring: CE vs. ND	2.42	1.51, 3.84	14.6	<0.001
Outbreak occurring in Africa: CE vs. ND	3.50	2.12, 5.84	24.8	<0.001
Outbreak occurring in Middle East: CE vs. ND	1.54	0.31, 7.78	0.39	0.530
Outbreak occurring: Both vs. CE	0.72	0.41, 1.29	1.24	0.267
Outbreak occurring: Both vs. ND	1.74	1.15, 2.62	7.2	0.007
VPD vs NVPD outbreak: CE vs. ND	4.14	1.90, 9.43	13.6	<0.001
Excluding 2009 H1N1 outbreaks				
Outbreak occurring: CE vs ND	2.27	1.57, 3.25	20.3	<0.001
VPD vs NVPD outbreak: CE vs ND	2.91	1.29, 6.91	7.7	0.009

When analysis included only vaccine-preventable outbreaks, we found that OR of a vaccine-preventable outbreak occurring in a CE only versus an ND only was 4.14 (95% CL 1.90, 9.43) (Table 5). We found no significant regional variations. When we excluded the H1N1 outbreak, the OR of a vaccine-preventable outbreak occurring in a CE only versus an ND only was 2.91 (95% CL 1.29, 6.91) (Table 5).

## Discussion

Our findings indicate that the odds of an outbreak occurring are higher in a CE than in a ND. When an outbreak was associated with a CE, it had greater odds of being a vaccine-preventable outbreak. CEs were also highly associated with concurrent NDs: 71% of CEs had an associated ND (118 both among 167 CEs). When examining the overlap of CEs and NDs, it would be natural to assume that concurrent events would place populations at higher risk for outbreak. Interestingly, we did not observe this; one possible reason is that the CE and ND combined may actually drive more people across international borders in search of safety and resources unavailable at home, with any associated outbreak not being in the emergency affected country. Despite the potential for misclassification, when we examined all of the ORs, we found statistical evidence that CEs are the driving force behind increased odds of vaccine-preventable outbreaks: the OR for CEs vs. ND was greater than four.

One major pattern that emerged from these data was that developing countries were disproportionately affected by CEs and outbreaks, especially in Africa where 67% each of total CEs and ‘Both’ events (CE and ND) and 46% of all outbreaks occurred. The baseline risk of disease occurring in developing countries is high. While only 39% of globally reported outbreaks were VPDs, 70% of outbreaks reported in Africa were vaccine preventable, compared with Europe and North America where only 11% of reported outbreaks were VPDs, and those numbers came almost exclusively from the 2011 measles outbreak (data not shown).

The causes of morbidity and mortality in the early phase of emergencies are widely understood. This study sought to look at the larger implications of emergencies by examining all WHO reported infectious diseases outbreaks in a country during the allotted time. What emerged has implications both for public health interventions and for areas of future study. One of the most obvious public health implications is that increased vaccine coverage in developing countries prone to emergencies could reduce morbidity and mortality associated with CEs. While the WHO and UN Children’s Fund (UNICEF) Global Immunization Vision and Strategy (GIVS) campaign and the Global Alliance for Vaccines and Immunizations (GAVI) have made strides in increasing routine vaccine coverage worldwide, many countries involved in conflict have seen their healthcare infrastructure destroyed and vaccine coverage progressively dropping [19–21]. This loss of herd immunity has regional and global implications. The re-emergence of polio in Syria in 2013 and in Cameroon in 2014 brought the potential international impact into sharp focus. WHO recognized the risk: “The consequences of further international spread are particularly acute today given the large number of polio-free but conflict-torn and fragile States which have severely compromised routine immunization services and are at high risk of re-infection. Such States would experience extreme difficulty in mounting an effective response were wild poliovirus to be reintroduced” [22].

This raises the question of how to increase coverage in areas, which have little or no infrastructure, where conflict is ongoing, or where conditions may be too dangerous to implement vaccination campaigns. While this seems challenging, successful vaccination campaigns have been implemented during active conflict in Afghanistan, Peru, and Democratic Republic of Congo [23]. However, these campaigns are with risk, as seen in Pakistan, Afghanistan, and Nigeria where healthcare workers carrying out vaccination activities have been killed [24]. While CEs are true emergencies, we might anticipate them, while we cannot anticipate NDs. Conflict and instability can be brewing for months or years before erupting. Prioritizing adaptable healthcare infrastructure and vaccine coverage in high-risk countries could mitigate the negative disease outcomes when a CE occurs not only in the specific countries but also can improve the global health security overall.

This research has several limitations: first, we linked these data by year and country so we can infer no causal relationship. Second, events occurring in different regions within a country may be linked by inclusion criteria but may in actuality be unrelated. Third, some emergencies that occurred at the end of a year may be linked but recorded in different years and therefore not associated in the analysis. Fourth, for NDs, nine of the 10 years had multiple reports of events for the same country in the same year. If these were different types of disasters, they were each counted as a single occurrence. Because of this, the number of ‘Both’ events may be artificially inflated.

Furthermore, the databases may have reporting inconsistencies. We used multiple databases to try to avoid misclassification. The WHO archives are from individual countries, which may collect and report data differently by country. Some reports appeared to provide estimates or rounded numbers, while other reports had more precise data. Some reports do not include mortality data and do not always specify if case numbers are laboratory-confirmed cases or only symptomatically diagnosed. Additionally, no other database was available for outbreak verification. For all categories, events may also have been mis-categorized or missing. The various databases used may not have included all events.

Finally, while this research can give a broad picture of associations, many nuances of relationships are missed. One example is the cholera outbreak in Haiti after the 2010 earthquake. The epidemic was actually traced back to Nepalese UN Peacekeepers who brought the disease after the earthquake [25]. One could argue that the epidemic would not have occurred if the earthquake had not happened. However, the opposite could be argued as UN Peacekeepers had been in Haiti long before the earthquake occurred. The complexity of causality in these circumstances is not always clear.

## Conclusions

While examining these emergencies and outbreaks through a public health lens, primary prevention should always be the goal. But, as the International Committee of the Red Cross stated, “Disasters and emergencies are a fundamental part of normal life. They are consequences of the ways societies structure themselves, economically and socially; the ways that societies and states interact; and the ways that relationships between the decision makers are sustained.” [26] However, effective secondary prevention is possible. While it is beyond the scope of this research to predict the occurrence of natural disasters and humanitarian emergencies, these data may be used to mobilize future resources for mitigation, planning, and response strategies. These strategies should manage the consequence of disasters and emergencies and decrease the risk of epidemics, especially vaccine-preventable epidemics, and thereby improve overall global health security.

## Abbreviations

CE: Complex emergency
CE-DAT: CRED Emergency Event Database
CERF: Central Emergency Response Fund
CFR: Case fatality rate
CMR: Crude mortality rate
CRED: Center for Research on the Epidemiology of Disasters
EM-DAT: CRED International Disaster Database
MERS-CoV: Middle Eastern Respiratory Syndrome Coronavirus
ND: Natural disaster
OCHA: United Nations Office for the Coordination of Humanitarian Affairs
UNHCR: United Nations High Commissioner for Refugees
VPD: Vaccine-preventable disease
